# Deleterious Effects of Histidine Tagging to the SH3b Cell Wall-Binding Domain on Recombinant Endolysin Activity

**DOI:** 10.4014/jmb.2408.08003

**Published:** 2024-10-11

**Authors:** Jin-Mi Park, Jun-Hyun Kim, Kang-Seuk Choi, Hyuk-Joon Kwon

**Affiliations:** 1 Laboratory of Avian Diseases, Department of Farm Animal Medicine, College of Veterinary Medicine and BK21 PLUS for Veterinary Science, Seoul National University, Seoul 08826, Republic of Korea; 2 Laboratory of Poultry Medicine, Department of Farm Animal Medicine, College of Veterinary Medicine and BK21 PLUS for Veterinary Science, Seoul National University, Seoul 08826, Republic of Korea; 3 Research Institute for Veterinary Science, College of Veterinary Medicine, Seoul 08826, Republic of Korea; 4 Farm Animal Clinical Training and Research Center (FACTRC), GBST, Seoul National University, Gangwon-do 25354, Republic of Korea; 5 GeNiner Inc., Seoul 08826, Republic of Korea

**Keywords:** Antibacterial activity, cell-free expression system, chimeric lysins, *Staphylococcus aureus*, histidine tag, SH3b cell wall-binding domain

## Abstract

Natural and artificial endolysins exhibit bactericidal effects by destroying peptidoglycans in the cell wall of gram-positive bacteria and are usually composed of an N-terminal catalytic domain (CTD) and a C-terminal cell wall-binding domain (CBD). The structures and receptors of CBDs are variable, but bacterial Src homology 3 (SH3b) CBDs are prevalent among the natural endolysins of *Staphylococcus aureus*. Moreover, although recombinant endolysins with a C-terminal 6x histidine tag (His-tag) are often produced and convenient to purify, the deleterious effects of His-tags on antibacterial activity have not been evaluated thoroughly. Recently, we reported that the antibacterial activity of a commercial lysostaphin without a His-tag differed from that of cell-free lysostaphin with a C-terminal His-tag, and lysostaphin also contains a C-terminal SH3b CBD. In this study, we directly compared the effects of His-tags on the antibacterial activities of lysostaphin and several chimeric lysins possessing different SH3b CBDs. We confirmed that antibacterial activity decreased 16.0-32.0-fold after a His-tag was added to the SH3b CBD.

## Introduction

*Staphylococcus aureus* is a zoonotic and reverse zoonotic pathogen that causes various diseases in humans and animals [1]. Methicillin-resistant *S. aureus* (MRSA) has become a serious threat to human and animal health, and new antibacterial approaches are needed due to the increasing antibiotic resistance [2]. Natural endolysins derived from bacteriophages, which are usually composed of a modular catalytic domain (CTD) and a cell wall-binding domain (CBD), bind and selectively cleave covalent bonds between N-acetyl glucosamine (NAG) and N-acetyl muramic acid (NAM) di-unit glycan chains and/or stem and cross-linking pentaglycine peptides of cell wall peptidoglycans (PGs) [3]. Artificial chimeric lysins have been successfully developed to overcome the narrow spectrum and/or low antibacterial activities of natural endolysins and offer promising alternatives to conventional antibiotics [4].

The binding between eukaryotic SH3 (Src homology 3) domains and proline-rich peptides influences cell-cell communication and intracellular signaling from the cell surface to the nucleus, and bacterial SH3 (SH3b) occurs in the CBD of phage endolysins [5]. To date, approximately 65.5% (19/29) of recombinant endolysins that are active against *S. aureus* have been produced with a C-terminal 6x histidine tag (His-tag); through this process, endolysins can be conveniently purified from *E. coli*. Among them, 44.8% (13/29) of the recombinant endolysins contain a SH3b CBD, and His-tags were attached to this structure [6]. Due to the prevalence of SH3b CBDs among natural phage endolysins, researchers have recently developed SH3b CBDs with a His-tag to generate very active recombinant endolysins [7, 8].

His-tags are widely used in research and industrial applications because of their small size and low immunogenicity; however, the effects of His-tags on the functional properties of proteins, including chimeric lysins, are not fully understood [9, 10]. While some researchers have reported that His-tags have negligible effects on protein structure, activity, or molecular interactions, other studies have reported the opposite [10]. Immobilized metal affinity chromatography (IMAC) can be used to effectively isolate His-tagged proteins from biological mixtures because of the affinity between the His-tag and nickel [11]. However, compared with ion exchange chromatography, these methods are more expensive, and there is a risk of contamination by toxic metal ions during purification [12]. Commercial lysostaphin (Sigma Aldrich; L9043) is purified by cation exchange chromatography because it has no His-tag, and this lysostaphin exhibited superior antibacterial activity to His-tagged lysostaphin expressed in a cell-free expression system in our previous study [13]. In addition, this lysostaphin possesses a SH3b CBD [14].

Previously, we improved the *E. coli* cell-free expression system to generate chimeric lysins in a simple and rapid manner and compared their antibacterial activities [13, 15]. ClyC and ClyO are chimeric lysins composed of a LysSA12 CHAP domain and a LysPALS1 CBD, as well as a CHAP domain from *S. aureus* and a CBD from *Staphylococcus suis*-specific PlySs2 phage endolysins, respectively [8, 16]. Additionally, we reported the notable antibacterial activity of Lsp-ClyC_SH3_, which is a chimeric lysin composed of the M23 peptidase domain of lysostaphin and the CBD of ClyC [13]. In this study, we directly compared the effects of His-tags on the antibacterial activities of lysostaphin, ClyC, ClyO, and Lsp-ClyC_SH3_, all of which possess different types of SH3b CBDs [13]. The antibacterial activities of the tested endolysins without His-tags consistently increased compared to those of the endolysins with His-tags, and the antibacterial activities of lysostaphin and Lsp-ClyC_SH3_ increased greatly (approximately 16.0- to 32.0-fold). We propose that steric hindrance of the His-tag with the glycan chains of PG likely results in a reduction in SH3b CBD binding to the target stem and cross-linking peptides [14, 17, 18].

## Materials and Methods

### Bacterial Strains

MRSA strains that cause infections in humans, CCARM3806, CCARM3825, CCARM3832, and CCARM3837, were used and cultivated as previously described [13].

### Preparation of Chimeric Lysin Templates and Mutagenesis

To generate a His-tag (24 nucleotides, AAG GGT CAT CAT CAC CAT CAC CAT, encoding KGHHHHHH) deleted coding fragment for cell-free expression, we used the primer sets listed in Table 1: Lsp-dHis, ClyC-dHis, and ClyO-dHis [13]. The amplified products were then assembled with T7 promoter/T7 terminator fragments by splice overlap extension PCR (SOE-PCR) as previously described [19].

ClyC-L25 was generated by deleting 20 amino acids (154-173; ETAPR SVQSP TQASK KETVD) from the ClyC linker with the Phusion Site-Directed Mutagenesis Kit (Thermo Fisher Scientific Inc., USA) and 5'-phosphorylated ClyC-L25 primer sets [8]. The D74, E101, and D105 residues of the cysteine, histidine-dependent amidohydrolases/peptidases (CHAP) domain of ClyC were mutated to glycine with a Muta-Direct Site Directed Mutagenesis Kit (iNtRON Biotechnology, Republic of Korea) with the primer sets D74G, E101G and D105G (Table 1). Subsequent SOE-PCRs with the T7 promoter/T7 terminator fragments were performed as previously described [13].

### Cell-Free Expression and Antibacterial Activity Test

We previously described a method for producing cell-free synthetic proteins and implementing a rapid screening test [13]. In this study, proteins were synthesized and the antibacterial activities of the chimeric lysins were assessed following the previously described methods [13]. Briefly, single bacterial colonies were cultured overnight in TSB at 200 rpm in a 37°C incubator. The grown MRSA strains were diluted to 2 × 10^7^ CFU/ml with TBS, and 100 μl was added to a 96-well plate. Varying amounts of protein, ranging from 1 μl to 0.015 μl by twofold dilution, were mixed with the bacteria. The mixture was incubated at 37°C with shaking at 200 rpm for 6 h, and the OD_600_ value was observed every hour.

### Comparison of Expressed Protein Levels

To generate biotinylated lysine residues, we utilized the manufacturer's protocol for the cell-free synthesis system with a slight modification. Instead of adding 3 μl of DNase-free distilled water to the protein synthesis mixture, we used 3 μl of Transcend tRNA (Promega Corp., USA; L5061). For western blot analysis, 10 μl of the protein supernatant was mixed with 50 μl of acetone and incubated for 15 min on ice. This mixture was then centrifuged at 12,000 ×*g* for 5 min to remove the supernatant, after which the pellet was dried. The pellet was resuspended in 20 μl of RIPA lysis and extraction buffer (Thermo Fisher Scientific Inc.). Subsequently, 5 μl of 4 × SDS sample buffer was added, and the mixture was vortexed and boiled at 100°C for 5 min. After being subjected to SDS-PAGE, the proteins were transferred to a nitrocellulose membrane and blocked with TBST for 1 h. Streptavidin-HRP (5 μl, Invitrogen) was mixed with 10 ml of TBST and incubated for 1 h. The relative intensity of the protein bands was quantified by ImageJ software (ver 1.5.3; NIH, USA).

### In Silico 3D Structure Prediction

The protein structure was predicted with AlphaFold2 and visualized with UCSF ChimeraX. The binding between CBD and pentaglycine (PDB: 5LEO) was modeled with HPEPDOCK 2.0.

## Results

### Minor Effects of His-Tags on Production of Recombinant Proteins in a Cell-Free Expression System

**Fig. 1:**
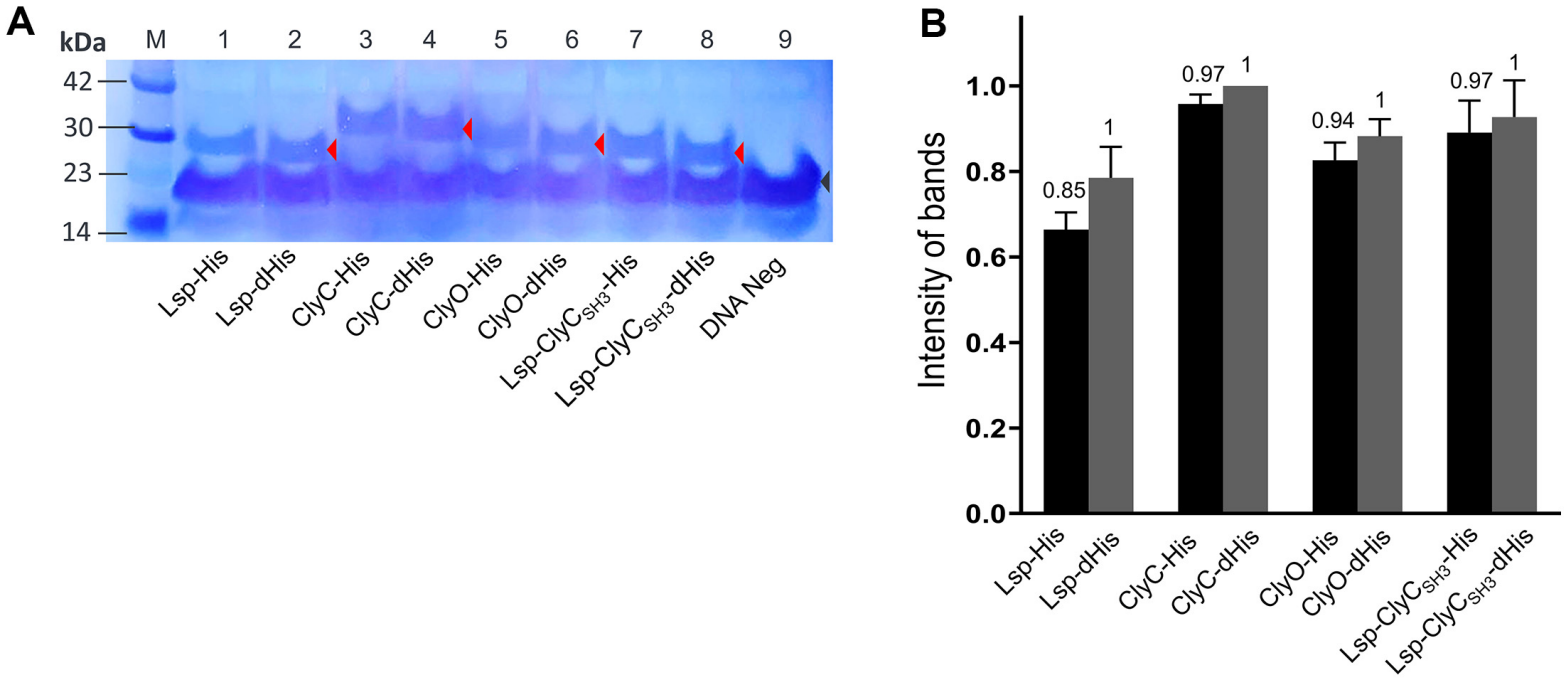
Cell-free expression and quantification of recombinant proteins with and without His-tags. (**A**) Western blot analysis of chimeric lysins labeled with biotinylated lysine and detected with HRP-streptavidin. Lane 1: Lsp-His (28.1 kDa); Lane 2: Lsp-dHis (27.1 kDa); Lane 3: ClyC-His (32.1 kDa); Lane 4: ClyC-dHis (31.1 kDa); Lane 5: ClyO-His (29.6 kDa); Lane 6: ClyO-dHis (28.5 kDa); Lane 7: Lsp-ClyC_SH3_-His (28.1 kDa); Lane 8: Lsp-ClyC_SH3_-dHis (27.1 kDa); Lane 9: DNA negative sample. The red and black arrows indicate the bands of the expected proteins and the bands of innate BCCP in the *E. coli* S30 extract. (**B**) The relative intensity of the protein bands was quantified with ImageJ (ver. 1.53). The experiment was performed in triplicate and the mean ± SD values are shown. The ratios of the protein amounts of recombinant proteins with (black bar) and without (gray bar) His-tags are represented on the top of each bar.

We labeled the recombinant proteins with a commercial biotinylated lysine that is incorporated into the AAA codon-binding tRNA. The number of AAA codons per recombinant protein differs, and 14, 12, 13, and 11 AAA codons are present in lysostaphin (Lsp), ClyC, ClyO, and Lsp-ClyC_SH3_, respectively [8, 12, 13, 16]. The recombinant proteins produced enough strong bands for comparison after western blotting (Fig. 1A), and the densities of the specific bands were measured (Fig. 1B). Considering the presence of the biotinylated protein, biotin carboxyl carrier protein (BCCP), the strong band between the 14-23 kDa molecular weight markers may be related to BCCP in the *E. coli* S30 extract of a cell-free expression reaction [20]. The relative amount (amount ratio) of each recombinant protein with a His-tag to that without a His-tag is represented on top of the bar (Fig. 1B). Interestingly, all the densities of the His-tagged recombinant proteins were slightly lower than those of the corresponding recombinant proteins without His-tags, but the amounts of recombinant proteins with or without His-tags differed by 15% (Lsp) at most.

### Deleterious Effects of His-Tags on the Antibacterial Activities of Lysostaphin and Chimeric Lysins

**Fig. 2:**
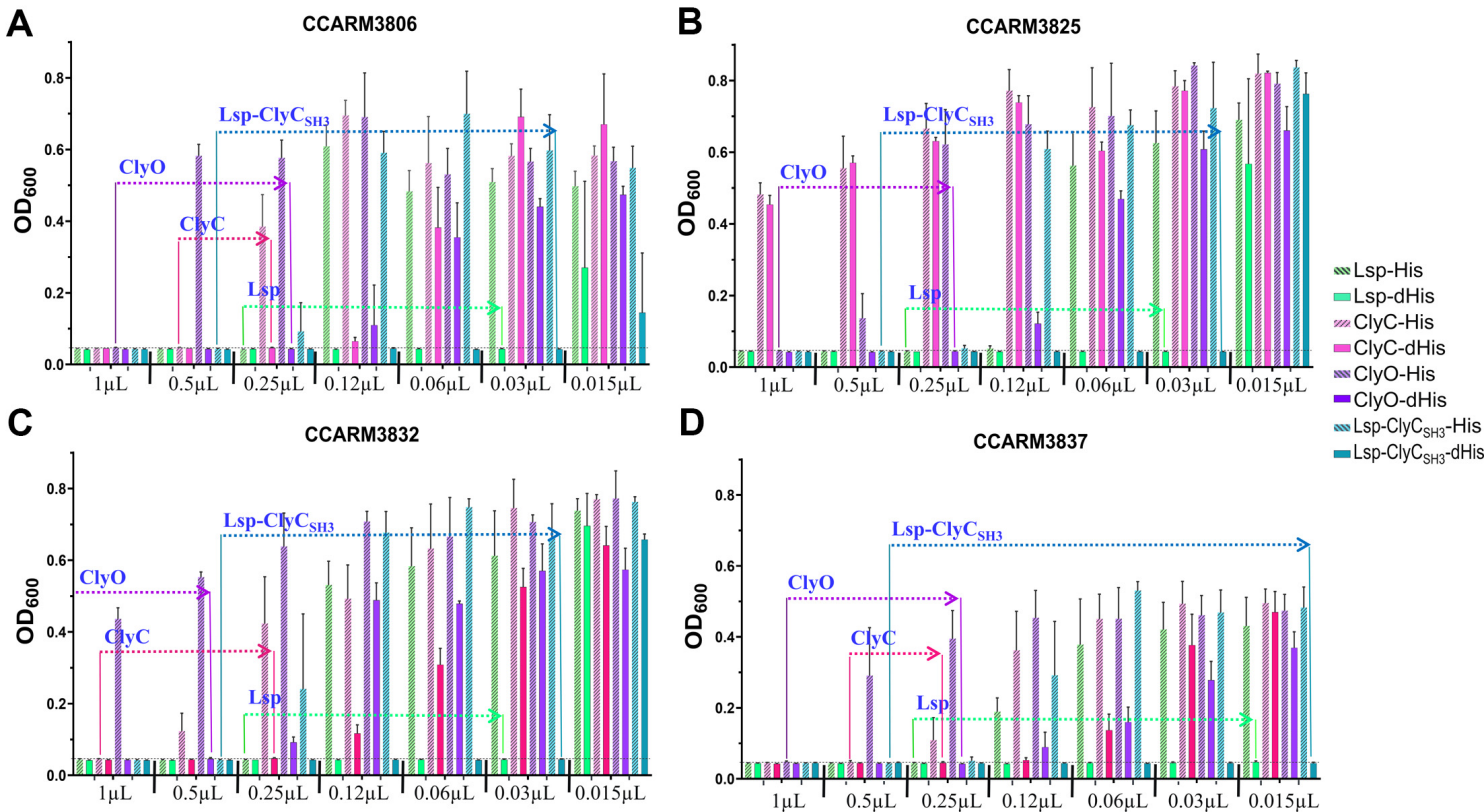
Antibacterial activities of lysostaphin and chimeric lysins with and without His-tags. The antibacterial activity of the cell-free expressed lysostaphin and chimeric lysins was tested with a rapid screening test against the following MRSA strains that cause infections in humans: (**A**) CCARM3806, (**B**) CCARM3825, (**C**) CCARM3832, and (**D**) CCARM3837. The recombinant proteins were diluted twofold, ranging from 1 to 0.015 μl, and the ratios were multiplied to adjust the different amounts of recombinant proteins with and without the His-tag. The fold difference of each recombinant protein between the minimal antibacterial volumes is represented in red. All the experiments were performed in triplicate, and the results are presented as the means ± SDs. In the area below the dotted line, no *S. aureus* growth occurred during the 6-h reaction.

The cell-free expressed recombinant proteins that were diluted twofold were added to the four MRSA strains, and their antibacterial activities were determined with the minimal volume (μl) of the original solution of recombinant proteins (Fig. 2). Owing to the different amounts of recombinant proteins with and without His-tags, the ratios were multiplied by the minimal volume of His-tagged recombinant protein. Lsp-His showed antibacterial activity at 0.21 μl (0.25 μl × 0.85) for all the tested strains, but Lsp-dHis exhibited antibacterial activity at 0.015 μl for CCARM3837 and 0.03 μl for the other strains. The antibacterial activity of lysostaphin increased by 14.0- and 7.0-fold after the His-tag was removed. Similarly, after the His-tag of ClyC was removed, the antibacterial activities of CCARM3806, CCARM3832, and CCARM3837 increased by 1.9-, 3.9- and 1.9-fold, respectively. ClyC-His and ClyC-dHis did not show antibacterial activity toward CCARM3825. The removal of the ClyO His-tag increased the antibacterial activity over 4.0-fold for CCARM3832 and 3.8-fold for the other compounds. The removal of the Lsp-ClyC_SH3_ His-tag increased the antibacterial activity by 32.0-fold for CCARM3837 and 16.0-fold for the other strains (Table 2).

### Comparison of the In Silico 3D Structures of Lysostaphin and Chimeric Lysins with and Without His-Tags

**Fig. 3:**
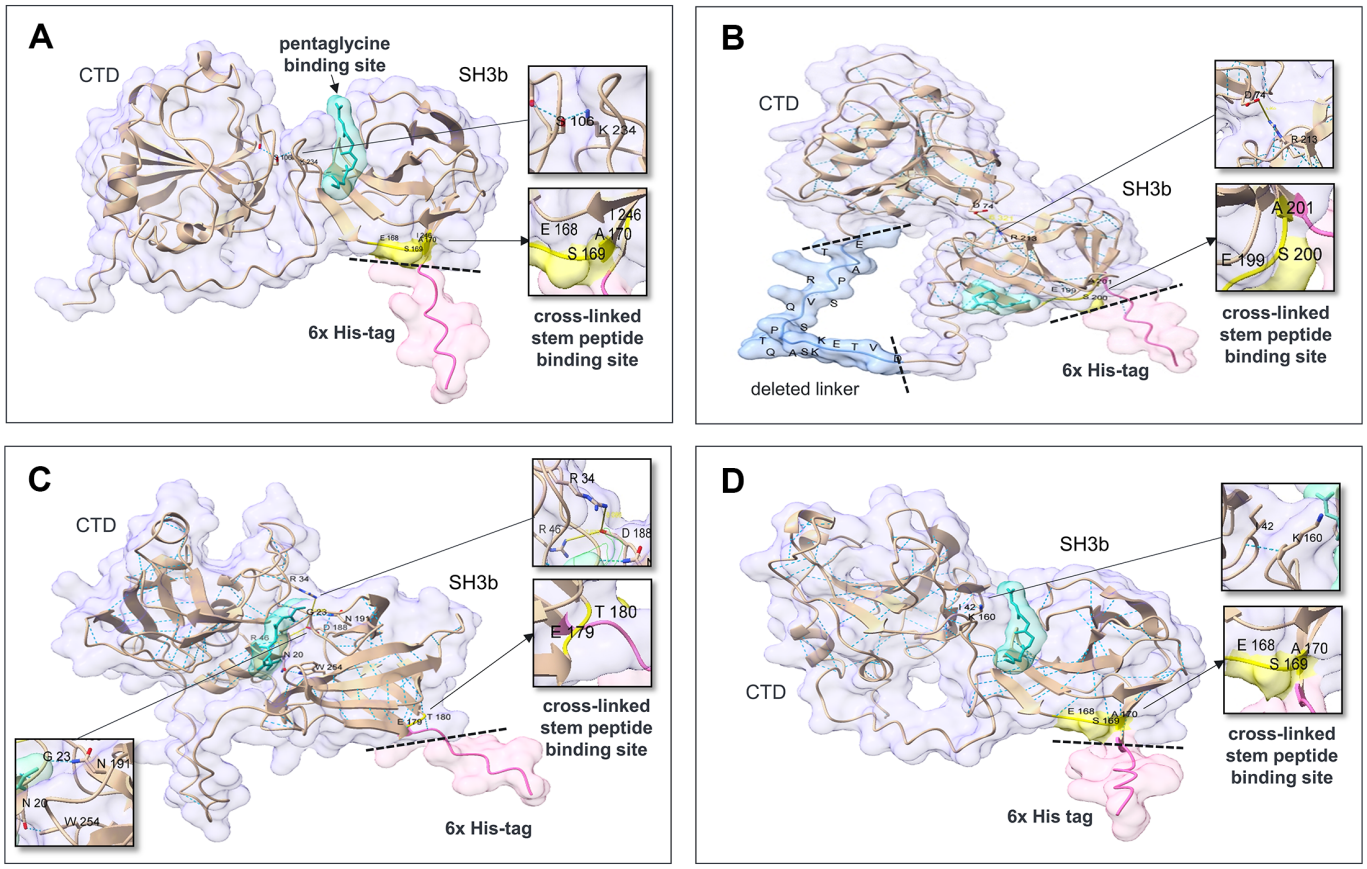
Effects of His-tags on the structures of lysostaphin and chimeric lysins. Structure and interdomain interactions were predicted for (**A**) Lsp-His, which formed a hydrogen bond between S106 and K234; (**B**) ClyC-His, which formed an electrostatic bond between D74 and R213 (6.321 Å); (**C**) ClyO-His, which formed a hydrogen bond between N20 and W254 and electrostatic bonds between R34 and D188 (8.086 Å) and R46 (7.975 Å); and (**D**) Lsp-ClyC_SH3_-His, which formed a hydrogen bond between I42 and K160. All the structures were predicted with AlphaFold2. The interaction between the cell wall-binding domain and pentaglycine was predicted with HPEPDOCK 2.0, with pentaglycine shown in green. The crosslinked stem peptide binding site and the region adjacent to the His-tag are colored in yellow. The His-tag located at the Cterminus of each protein is highlighted in pink. The dotted lines indicate the sites where the His-tags were removed.

To determine the effects of His-tags on protein structure and PG binding, we predicted the 3D structures of lysostaphin (Accession nos. AAB53783.1; 248-493) and chimeric lysins with and without His-tags with AlphaFold (Fig. 3) [17]. The reported pentaglycine site in the CBD of lysostaphin is far from the His-tag, but the stem peptide binding sites (the original residues E414, S415, and A416 correspond to E168, S169, and A170, respectively, in Fig. 3A) are near the His-tag [14, 21]. Therefore, owing to the steric hindrance of the glycan chain covalently linked to the stem peptide, the protruding His-tag may interfere with lysostaphin binding to stem peptides. The pentaglycine-binding sites of the ClyC and ClyO CBDs were predicted with HPEPDOCK 2.0 in silico, but the binding sites of these compounds to stem peptides were unclear because of amino acid differences. However, the similarity of the amino acid sequences and lengths of the CBDs of lysostaphin and ClyC may support the inference that His-tags exert similar effects on ClyC and Lsp-ClyC_SH3_, as they do on lysostaphin. Additionally, a more globular shape without His-tags may help promote more efficient binding/cleavage and movement in highly cross-linked, multilayered PG networks [22]. The linker length of ClyC is 45 amino acids, and it protrudes to likely destroy the globular morphology as well as the His-tag (Fig. 3B).

Although the predicted 3D structures revealed that the CTD and CBD were in close contact, lysostaphin did not provide a stable interaction interface between these domains [21]. In the predicted 3D structures of Lsp-His and Lsp-ClyC_SH3_-His, an interdomain hydrogen bond of S106 and I42 in the CTD with K234 and K160 in the CBD was predicted (Fig. 3A, 3D), but the 3D structures of Lsp-dHis and Lsp-ClyC_SH3_-dHis revealed that the spatial separation between the domains without His-tags was increased. For ClyC-His and ClyO-His, the electrostatic interdomain interactions of D74 and R34 in the CTD with R213 and D188 in the CBDs, respectively, as well as hydrogen bonds (N20 and W254 for ClyO) were predicted (Fig. 3B, 3C). The 3D structures of ClyO-dHis showed apparent separation of the domains and broken interactions without His-tags. In contrast, ClyC-dHis maintained the hydrogen bonds.

### Effects of Long Linker and Interdomain Interactions on the Antibacterial Activity of ClyC with and Without His-Tags

Linker optimization of chimeric lysins increases antibacterial activity, and we reduced the length of the linker of ClyC from 45 to 25 amino acids by removing the middle 20 amino acids (Fig. 3B) [8]. The 3D structure of the ClyC mutant (ClyC-L25) was rounder in shape without protruding into the angular region. His-tag removal increased the antibacterial activity of ClyC-L25-dHis by 5.0-fold for CCARM3806 and CCARM3837, and 4.8-fold for CCARM3832 (Table 3). Therefore, the shortened linker increased the fold difference in the antibacterial activities of ClyC-L25-dHis for CCARM3806 (5.0 vs. 1.9) and CCARM3837 (5.0 vs. 1.9) in comparison with ClyC.

According to the 3D structure prediction, the positive charges of R213 and H244 in the CBD of ClyC and the negative charges of D74, E101, and D105 in the CTD are in close proximity. To eliminate possible interactions, we mutated the acidic amino acids in the CTD to glycines in a stepwise manner. ClyC-D74G, ClyC-E101G, ClyC-E101G-D105G and ClyC-D74G-E101G-D105G were expressed, and the amounts and antibacterial activities of the recombinant proteins with and without His-tags were compared as described above (Table 3). Owing to its consistent resistance, CCARM3825 was not tested. Deleterious effects of the His-tags were observed in all the ClyC mutants, but the extent of the effects varied. The D74G mutation decreased the antibacterial activities of ClyC-D74G and ClyC-D74G-E101G-D105G with and without His-tags. Interestingly, the E101G and E101G-D105G mutations increased the fold differences in the antibacterial activities of ClyC-E101G-dHis for CCARM3837 (4.5 vs. 1.9) and of ClyC-E101G-D105G-dHis for CCARM3806 (3.9 vs. 1.9) and CCARM3837 (7.8 vs. 1.9) compared with those of ClyC.

## Discussion

The PG cell wall of *S. aureus* is multilayered and up to 20-40 nm thick. [18]. Glycan chains are composed of mainly 6 or 12 repeating units of NAG and NAM, and the four di-glycan units rotate right-handedly in a perpendicular direction at approximately 360° [22]. The perpendicular stem peptides attached to NAM are cross-linked to the stem peptides of neighboring glycan chains via pentaglycine. Cross-linking occurs between the fourth D-Ala residue of the cross-linked stem peptide and the third L-Lys residue of the bridge-linked stem peptide [23]. The cross-linking rate (74-92%) of *S. aureus* PG is relatively high, and the pore size may be smaller than that of other materials, which can accommodate spherical molecules up to 22 kDa when relaxed, and 50 kDa when stretched [23]. The CBD of lysostaphin binds to both pentaglycine and cross-linked stem peptides, but the binding affinity of lysostaphin for cross-linked stem peptides is much greater than that for pentaglycine [21]. The C-terminal His-tag does not interfere with the binding site for pentaglycine but is located close to the cross-linked stem peptide-binding sites composed of the residues E168, S169, and A170, as shown in Fig. 3A. Therefore, the C-terminal His-tag of lysostaphin may not fit due to steric hindrance with glycan chains bearing cross-linked stem peptides, possibly resulting in decreased antibacterial activity [21].

The SH3b domains are most prevalent among *Staphylococcus* and *Streptococcus* phage endolysins, and were possibly selected during the evolution of phages [24]. Although the CBDs of lysostaphin, ClyC, and ClyO are classified as SH3b, they are divided into different conserved domains, cl17036, pfam08460, and smart00287, respectively [24]. Among the MRSA genome sequences, the frequency of pfam08460 (38.2%) is greater than that of smart00287 (33.8%) and cl17036 (0.6%) [25]. The accurate binding sites of ClyC (pfam08460) and ClyO (smart00287) have not been determined, but the presence of similar amino acids (E199, S200, and A201 for ClyC; E179 and T180 for ClyO) corresponding to the cross-linked stem peptide-binding region of lysostaphin (E168, S169, and A170 as shown in Fig. 3A) and the large decrease in antibacterial activity resulting from the His-tag may support a binding mechanism similar to that of lysostaphin. The deleterious effect of the His-tag may be restricted to the SH3b domain, and no difference in antibacterial activity was observed between the non-SH3b domain-containing ClyH and ClyH with and without the C-terminal His-tag [26]. To date, researchers have reported various CBDs for which binding sites have not been identified, and intentional C-terminal tagging and testing changes in antibacterial activity may help clarify the receptors of CBDs in PG [6, 25].

In contrast to natural endolysins, linker optimization may be important for chimeric lysin activity. The different increases in antibacterial activity observed between ClyC-L25-His and ClyC-L25-dHis indirectly reflect the deleterious effect of the artificial long linker of ClyC; these effects result from the restriction of shape and size in the highly cross-linked PG of *S. aureus* [27]. Additionally, interdomain interactions may occur when CTDs and CBDs of different origins are combined artificially to generate chimeric lysins, which may also affect antibacterial activity. Notably, the isoelectric points of the ClyC CTD and CBD are acidic (pI 5.0) and basic (pI 8.0). In contrast, both lysostaphin and ClyO CTDs (pI 8.9 and pI 0.2, respectively) and CBDs (pI 9.7 and pI 8.9, respectively) are basic. Thus, interdomain interactions should be predicted and optimized during the development of chimeric lysin.

Lsp-ClyC_SH3_ exhibited greater antibacterial activity than did ClyC, which supports the importance of CTD. The reasons why Korean MRSA strains show different susceptibilities to the CHAP of ClyC and the endopeptidase of lysostaphin should be determined in further studies. The antibacterial activity of Lsp-ClyC_SH3_ is comparable to that of lysostaphin, and *S. aureus* strains isolated from cases of bovine mastitis in Korea are susceptible to lysostaphin [13]. The CBD of lysostaphin contains a T-cell epitope that induces strong humoral immunity and an N-linked glycosylation site that reduces antibacterial activity when expressed in yeast [28, 29]. The CBD of ClyC is different from that of lysostaphin in terms of its amino acid identity (49.5%) and does not contain an N-linked glycosylation site. Therefore, Lsp-ClyC_SH3_-dHis may be useful for clinical application.

In conclusion, we confirmed that adding a His-tag to SH3b domains negatively affects the antibacterial activity of chimeric lysins and that the linker length and interdomain interactions of chimeric lysins should be optimized.

## Figures and Tables

**Table 1:** PCR primers used for the amplification of His-tag-deleted fragments and ClyC mutants.

Primer name		Sequence (5’-3’)
Lsp-dHis	F	GTTCT GTGGG GTACT ATCAA A TGAG TTTAA ACTAT ATAG
	R	CTATA TAGTT TAAAC TCATT TGATA GTACC CCACA GAAC
ClyC-dHis	F	GCCTG GGGAA CATTC AAG TGAG TTTAA ACTAT ATAG
	R	CTATA TAGTT TAAAC TCACT TGAAT GTTCC CCAGG C
ClyO-dHis	F	CAATG CTTGG GGTAC ATTTAAA TGAG TTTAA ACTAT ATAG
	R	CTATA TAGTT TAAAC TCATT TAAAT GTACC CCAAG CATTG
ClyC-L25	F	AGTGCAAGTACACCGGCAACTAGACC\AGTTACAGGTTCTTG
	R	GCTTTTGAAGTTAGGACGGATAAACCACATAGGGAAGTCG
D74G	F	GTATACCAAA ATACACCAGGCTTCTTAGCGCAACCTGGC
	R	GCCAGGTTGCGCTAAGAAGCCTGGTGTATTTTGGTATAC
E101G	F	GTCATGTTGCATGGGTAATTGGTGCAACTTTAGATTATATCATTG
	R	CAATGATATAATCTAAAGTTGCACCAATTACCCATGCAACATGAC
D105G	F	GGGTAATTGGTGCAACTTTAGGCTATATCATTGTATATGAGCAG
	R	CTGCTCATATACAATGATATAGCCTAAAGTTGCACCAATTACCCA

**Table 2:** Comparison of the antibacterial activities of lysostaphin and chimeric lysins with and without His-tags.

Protein name	Increases in antibacterial activity (with His/without His)			
	CCARM3806	CCARM3825	CCARM3832	CCARM3837
Lsp	7.0-fold (0.21/0.03)^a^	7.0-fold (0.21/0.03)	7.0-fold (0.21/0.03)^a^	14.0-fold (0.21/0.015)
ClyC	1.9-fold (0.48/0.25)	NT^b^	3.9-fold (0.97/0.25)	1.9-fold (0.48/0.25)
ClyO	3.8-fold (0.94/0.25)	3.8-fold (0.94/0.25)	>4.0-fold (>1.0/0.50)	3.8-fold (0.94/0.25)
Lsp-ClyC_SH3_	16.0-fold (0.48/0.03)	16.0-fold (0.48/0.03)	16.0-fold (0.48/0.03)	32.0-fold (0.48/0.015)

^a^ Minimal antibacterial volume (μl) of recombinant proteins with and without His-tags. The volume was adjusted to the ratio measured by western blotting and densitometry. ^b^ Not measured at this concentration.

**Table 3:** Antibacterial activity comparisons of ClyC mutants with and without His-tags.

Mutated ClyC	Increases in antibacterial activity (with His/without His)		
	CCARM3806	CCARM3832	CCARM3837
ClyC-L25	5.0-fold (0.5/0.10)^a^	4.8-fold (1/0.21)	5.0-fold (0.5/0.10)
ClyC-D74G	2.0-fold (1/0.48)	>1.0-fold (>1/0.96)	2.0-fold (1/0.48)
ClyC-E101G	2.2-fold (0.5/0.23)	4.3-fold (1/0.23)	4.5-fold (0.5/0.11)
ClyC-E101G-D105G	3.9-fold (0.47/0.12)	3.8-fold (0.94/0.25)	7.8-fold (0.94/0.12)
ClyC-D74G-E101G-D105G	2.8-fold (1/0.36)	>2.8-fold (>1/0.36)	2.8-fold (1/0.36)

^a^Minimal antibacterial volume (μl) of recombinant proteins with and without His-tags. The volume was adjusted to the ratio measured by Western blotting and densitometry.
